# The Impact of Heat Exposure on the Health and Performance of Soccer Players: A Narrative Review and Bibliometric Analysis

**DOI:** 10.3390/sports12090249

**Published:** 2024-09-10

**Authors:** Spyridon Plakias, Themistoklis Tsatalas, Minas A. Mina, Christos Kokkotis, Andreas D. Flouris, Giannis Giakas

**Affiliations:** 1 Department of Physical Education and Sport Science, University of Thessaly, Karyes, 42100 Trikala, Greece; spyros_plakias@yahoo.gr (S.P.); ttsatalas@uth.gr (T.T.); 2 Department of Sport, Outdoor and Exercise Science, School of Human Sciences & Human Sciences Research Centre, University of Derby, Kedleston Road, Derby DE22 1GB, UK; m.mina@derby.ac.uk; 3 Department of Physical Education and Sport Science, Democritus University of Thrace, 69100 Komotini, Greece; ckokkoti@affil.duth.gr; 4 FAME Laboratory, Department of Exercise Science, University of Thessaly, 42100 Trikala, Greece; andreasflouris@gmail.com

**Keywords:** heat, football, hot weather, performance analysis, science mapping, clustering

## Abstract

The impact of heat exposure on the health and performance of soccer players is a widely discussed topic. The purpose of this study is to provide a comprehensive overview of the international literature that has addressed this issue. To achieve this objective, we initially conducted a bibliometric analysis and a literature review of the main topics that emerged through bibliometric techniques. For the bibliometric analysis, we employed VOSviewer software (version 1.6.20.0) and used documents found in the Scopus database. The analysis ultimately included 133 documents published in 66 sources. Key journals and authors were identified, highlighting significant contributions to the field. Science mapping revealed collaboration networks and research focus areas such as physical health, safety, soccer performance, dehydration and hydration, physiological mechanisms and monitoring, nutrition, fluid intake, and cooling techniques. Based on the key areas highlighted in the identified clusters, which emerged from the co-occurrence analysis of the author keywords, the following three topics were developed in the literature review: (a) the physiology and health of football players; (b) performance impacts; and (c) strategies to prevent negative consequences. The review showed that high heat exposure can reduce the physical and cognitive performance of athletes and prove detrimental to their health. To mitigate the negative consequences, appropriate hydration strategies, heat acclimatization, and cooling techniques have been proposed. Our findings provide the international scientific community with comprehensive knowledge of the existing literature, laying the foundation for future research while simultaneously offering coaches and athletes the necessary theoretical knowledge to help improve safety and performance.

## 1. Introduction

Soccer is an intermittent sport [1,2] in which players typically cover a total distance of 9 to 14 km during a 90 min period [3]. Between 22 and 24% of the total match distance is covered at speeds exceeding 15 km/h, 8–9% at speeds higher than 20 km/h, and 2–3% at speeds greater than 25 km/h [4]. Throughout a game, players can execute over 700 changes in direction and engage in approximately 1200 to 1400 activity shifts [4]. In terms of acceleration and deceleration, professional European soccer players carry out about 656 ± 57 accelerations and 612 ± 59 decelerations per match [4]. Beyond these physical demands, soccer players require focus, perception, anticipation, and quick decision-making [5,6].

A key challenge for soccer players is that matches are played in open stadiums where environmental conditions are uncontrolled, often resulting in high heat exposure. The body responds to these conditions by increasing sweating to maintain a stable body temperature, which is crucial for normal functioning [7,8]. However, excessive sweating without adequate fluid replacement can lead to adverse effects on both physical activity and cognitive function [9,10].

The impact of high heat exposure on athletic performance and health has become an increasingly important issue, especially in light of global climate change [11,12]. With rising temperatures, soccer players are more frequently exposed to heat stress, which can severely impair their performance and pose serious health risks. For example, several studies have documented that high temperatures negatively affect endurance, high-intensity actions, and cognitive function during matches [13]. Heat exposure has been shown to impair decision-making, reduce running distance, and increase physiological strain, such as higher heart rates and core temperatures [14,15]. Furthermore, the risk of heat-related illnesses, such as heat exhaustion and heat stroke, becomes a significant concern during games played in hot climates [11].

Elite soccer clubs have been consistently interested in having practical and usable models to protect the health of their players [16]. Understanding how high heat exposure affects soccer players is crucial for developing effective strategies to protect the players’ health and performance. This includes optimizing hydration practices, implementing heat acclimatization protocols, and utilizing cooling techniques during training and matches [17,18]. Additionally, exploring the physiological and biochemical responses to heat stress can help tailor specific interventions to enhance player safety and performance [19].

As a result, the main purpose of this study is to synthesize current research on the impact of high heat exposure on soccer players. We performed a bibliometric analysis of documents that examine the effect of heat exposure on soccer. Bibliometric analysis has become a well-established scientific method, particularly in the scientific and applied disciplines [20]. This method contributes to information about the literature in a given field [21]. In particular, bibliometric analysis aids in pinpointing significant publications, prominent researchers, and major institutions, as well as identifying emerging topics and areas where research is lacking. Bibliometric analysis encompasses both performance analysis and science mapping techniques [22]. Performance analysis involves calculating various metrics, such as the total number of publications, annual publication counts, and citation counts (by author, source, or organization). Science mapping techniques are used to detect connections and collaborations among researchers, scientific journals, and universities [23]. Techniques like co-authorship analysis, co-occurrence analysis, bibliographic coupling, and co-citation analysis are employed in science mapping [24]. Additionally, modern software like VOSviewer (version 1.6.20.0) allows for data visualization, which is beneficial for drawing conclusions and making informed decisions [25,26].

Although some review articles have been carried out on various topics related to heat exposure in soccer [27,28,29,30], a bibliometric analysis in this field is missing from the international literature. Therefore, to fill the gap in the existing literature and provide a comprehensive overview of the subject, we performed not only a bibliometric analysis but also a review of the main topics, which emerged through bibliometrics techniques (a. the physiology and health of football players; b. performance impacts; and c. strategies to prevent negative consequences). By doing so, we aim to provide the international scientific community with a comprehensive knowledge of the existing literature, laying the foundation for future research.

## 2. Material and Methods

The documents were searched in the Scopus database on May 30, 2024, using the BOOLEAN expression (TITLE (heat OR “hot weather” OR “high temperature” OR dehydration OR “sweat loss” OR “fluid loss” OR hydration OR “global warm” OR sweating) AND TITLE-ABS-KEY (soccer)). From the initial search, 149 documents were found. After reading the titles and abstracts, 16 documents were deemed unsuitable since they did not concern the relationship between heat exposure and soccer. The eligibility of the papers was coherently reviewed and agreed upon by the first two authors. All types of documents were accepted (research articles, review articles, books, editorials, etc.) written in any language and in any year, as long as their topic was related to heat exposure in soccer.

The CSV (Comma Separated Values) file obtained from Scopus was imported into VOSviewer (1.6.20.0) to apply the bibliometric analysis techniques mentioned earlier. Furthermore, after converting the file to an Excel (xls) format, it was imported into Microsoft Power BI for visualizing the documents per year. Power BI is a business intelligence tool tailored for visualizing statistical data [31,32].

For the bibliometric analysis, both performance analysis and science mapping were conducted. Regarding performance analysis, the number of documents per year, the sources with the most documents, and the authors with the most citations were calculated. The following science mapping techniques were used: (a) Co-authorship analysis with authors as the unit of analysis, which examines the collaborations between authors based on the documents they have co-authored; (b) Bibliographic coupling with sources as the unit of analysis, which examines the extent to which two or more sources cite common documents; (c) Co-citation analysis with authors as the unit of analysis, which analyzes the frequency with which two or more authors are cited together in other documents; and (d) Co-occurrence analysis with author keywords as the unit of analysis, which examines the frequency with which two or more keywords appear together in the same documents.

## 3. Bibliometric Analysis

### 3.1. Performance Analysis

**Figure 1:**
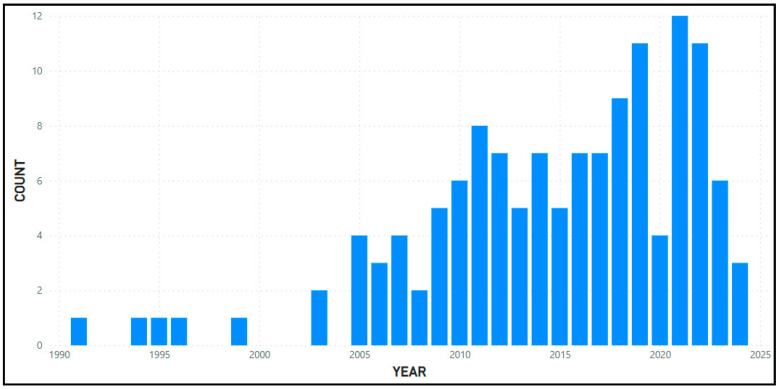
Number of documents per year.

A total of 133 documents were included in the bibliometric analysis. Figure 1 shows the number of documents per year, where it appears that the year 2021 was the one with the most (12).

The 133 documents have been published in 66 different sources, of which 10 have at least 5 publications (Table 1). A total of 530 authors have participated in the writing of 133 documents. Table 2 shows the top 20 authors in citations.

### 3.2. Science Mapping

**Figure 2:**
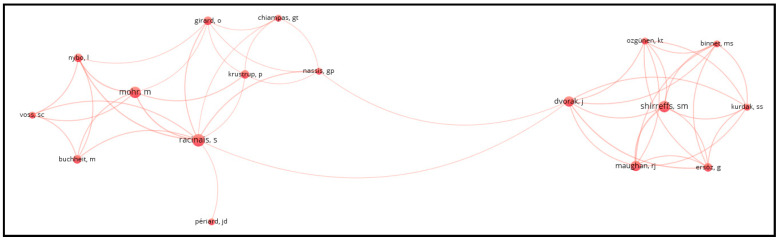
Co-authorship analysis with authors as the unit of analysis.

Figure 2 illustrates the co-authorship analysis with authors as the unit of analysis. This analysis employed two constraints: (a) only authors with at least two documents and (b) only authors who are connected to each other. The analysis reveals two main clusters of authors who collaborate closely. The connections between the authors indicate collaboration and the flow of information among various research groups. Authors such as Racinais, Shirreffs, Dvorak, and Nassis seem to have several collaborations within the network.

**Figure 3:**
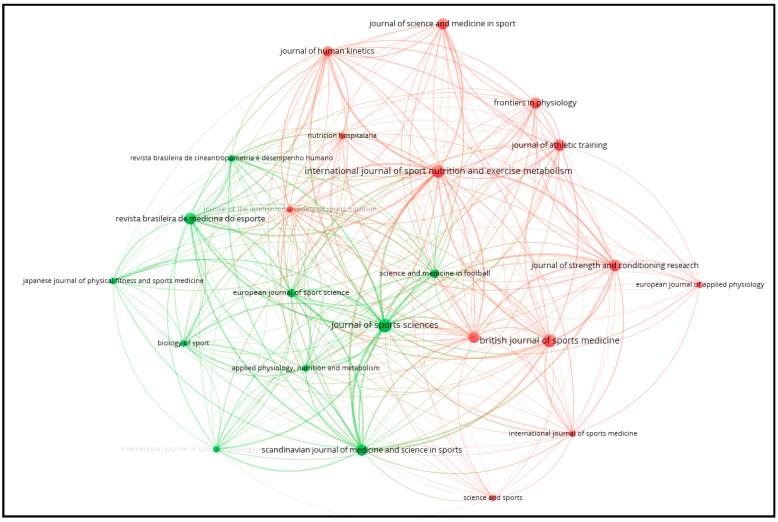
Bibliographic coupling with sources as the unit of analysis.

Figure 3 shows the bibliographic coupling network of sources. This analysis utilized two constraints: (a) only sources with at least two documents and (b) only sources that are connected to each other. The network visualization reveals how various academic journals are interlinked based on shared references. Prominent journals such as the Journal of Sports Sciences, the British Journal of Sports Medicine, and the International Journal of Sport Nutrition and Exercise Metabolism exhibit significant connections, indicating their influential role and frequent citation within this research area. The colors red and green in the network represent different clusters of journals that are closely related based on shared references, with each color indicating a distinct group of sources that exhibit stronger bibliographic coupling within their cluster.

**Figure 4:**
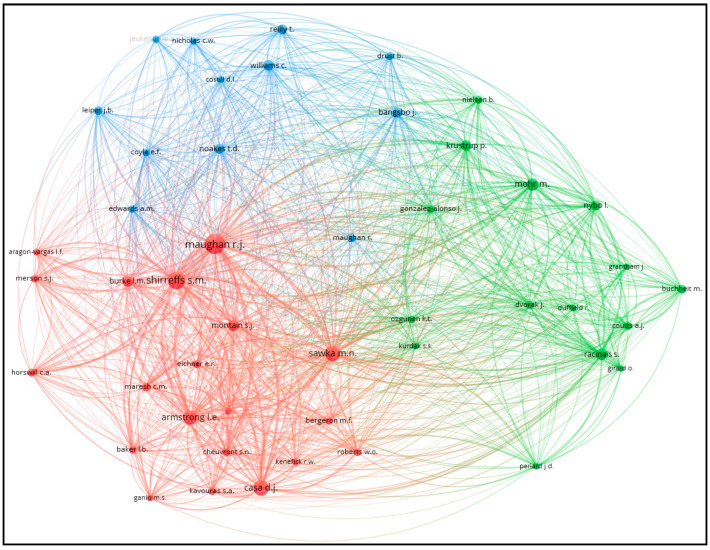
Co-citation analysis with authors as the unit of analysis.

Figure 4 presents the co-citation network of authors associated with research on soccer in high temperatures. This analysis was conducted using authors as the unit of analysis and included only those with at least 30 citations. The network visualization illustrates the relationships and frequency of co-citations among authors, highlighting key researchers who frequently appear together in the literature. Prominent authors such as Maughan R.J., Shirreffs S.M., and Mohr M. are central nodes in the network, indicating their significant influence and widespread recognition in this field of research. The different colors (red, blue, and green) represent distinct clusters of authors who are more frequently co-cited together, reflecting specific research areas or collaborations within the broader field.

**Figure 5:**
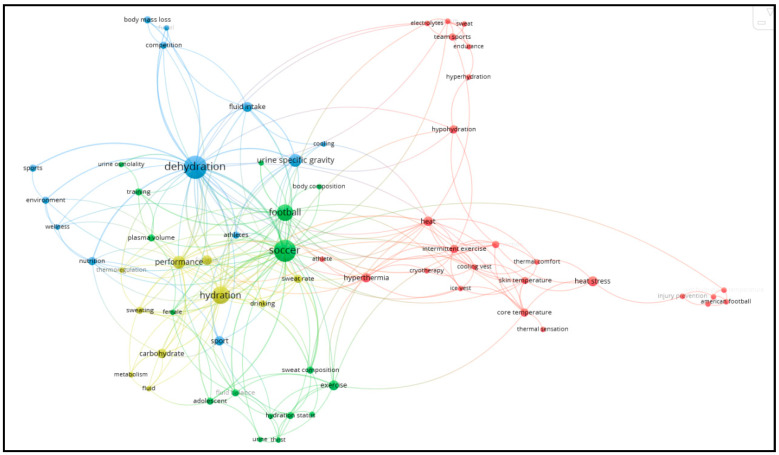
Co-occurrence analysis with author keywords as the unit of analysis.

From the co-occurrence analysis of the author keywords, the map in Figure 5 emerged, showing 4 clusters. The items in the red cluster pertain to physical health and safety, especially concerning heat and thermal conditions. The yellow cluster involves performance, hydration, and physiological mechanisms. The green cluster relates to monitoring physiological mechanisms in soccer. Finally, the blue cluster seems to focus on how various factors, such as nutrition, fluid intake, and cooling techniques, can affect athletes’ wellness and competitiveness, with dehydration being the central pillar. Based on the key areas highlighted in the identified clusters, the following three topics will be covered in the subsequent sections of the article: (a) the physiology and health of soccer players, (b) performance impacts, and (c) strategies to prevent negative consequences. Forty articles were used to conduct the literature review. The articles were selected based on their direct relevance to the three topics, ensuring that existing and up-to-date knowledge on these topic areas is comprehensively covered.

## 4. Literature Review

### 4.1. Changes in the Physiology and Health of Soccer Players

The physiological changes in soccer players during high-heat conditions are multifaceted. Core body temperature increases significantly during physical exercise, reaching 39–40 °C during a soccer game [33]. To prevent dangerous temperature increases, the body activates thermoregulatory mechanisms, primarily resulting in vasodilation of the skin’s blood vessels and increased sweating to cool the body through evaporation [34,35].

Sweat loss can range from 2 to 5% of a player’s body mass; however, with adequate fluid intake, the actual body mass loss is reduced to 1–3% [36]. The degree of sweating in the heat is approximately double that in moderate temperatures [37]. Greater dehydration occurs with double training sessions in a single day or pre-existing dehydration before the game [9]. Such conditions also lead to increased cortisol levels, adding stress to the body [10]. Sweat loss also varies by player position. Research by Djaoui et al. [36] shows that wide midfielders lose significantly more body mass than goalkeepers, central defenders, and forwards, while central midfielders lose more body mass than goalkeepers. Symptoms of dehydration in athletes include thirst, irritability, headache, weakness, vomiting, cramps, chills, nausea, and decreased performance [38].

Dehydration has significant consequences for the cardiovascular system. Heart rate increases due to sympathetic activation as venous blood return to the heart decreases, with blood vessels directing more blood to the skin [39]. Plasma volume also decreases, and dehydration from sweating causes changes in plasma osmolality and redox status [40]. Dehydration must be closely monitored, as it predisposes players to cardiac alterations during prolonged exercise in high-heat environments [9].

The central nervous system is crucial for exercise in the heat. Brain temperature exceeds the core temperature during prolonged exercise in the heat. Blood flow velocity in the cerebral artery decreases significantly in hyperthermia [41]. Dehydration reduces blood flow to the brain, leading to decreased cognitive performance and slowing information transmission in the prefrontal cortex [42]. Dehydration greater than 2% of body weight negatively affects attention, memory, and psychomotor activities [43]. During heat-induced fatigue, dopamine decreases and norepinephrine increases, causing lethargy and reduced motivation while reducing performance [44].

Loss of Na^+^ through sweat can cause plasma hyponatremia when [Na^+^] concentration falls below 130 mmol/L [38]. Hyponatremia worsens if fluids are replaced with only plain water during prolonged exercise in the heat [33,37]. The body responds by increasing antidiuretic hormone and aldosterone during exercise, particularly in dehydrated athletes. Aldosterone increase leads to lower [Na^+^] and higher [K^+^] in urine. This phenomenon is less intense in women [37]. Additionally, sweat loss estimation inaccuracies among players, noted by Davis et al. [45], highlight the risk of dehydration due to underestimating fluid needs.

Finally, high heat exposure also affects soccer players in other ways: increased muscle glycogen use [36], greater internal load despite the same external load [46], and increased cellular and oxidative stress [40]. Furthermore, exercise and heat stress generate free radicals that can damage leukocyte DNA, but heat shock protein 70 increases, offering protection against this damage [47].

### 4.2. Changes in Soccer Player Performance

As mentioned above, one of the body’s protective mechanisms against excessive increases in body temperature is increased sweating, which aims to cool the body through the evaporation of sweat [34,35]. Both physical exercise and high environmental temperatures increase the rate of sweating [48,49,50,51]. The combination of physical exercise with high heat exposure results in increased sweating to such an extent that, even with good hydration strategies, a reduction in body mass cannot be avoided [52]. The problem becomes even more significant with poor fluid replacement strategies during exercise and is exacerbated if the athlete is dehydrated to some degree before the start of exercise [53]. A body mass loss of less than 1% is considered not to affect soccer player performance [37]. However, dehydration levels greater than 1% are sufficient to reduce performance [33]. When dehydration reaches moderate levels (2%), which is very common in matches played in high temperatures, the reduction in performance becomes very significant [36]. Brocherie et al. [54] showed that the likelihood of favorable performance outcomes decreases by 3% for every 1 °C increase in environmental temperature for teams not acclimated to high temperatures.

There is a vast amount of research on the performance areas affected by high temperatures, resulting in significant differences in player performance and team outcomes. No and Kwak [39] and Edwards et al. [55] studied soccer players’ endurance under high-temperature conditions. No and Kwak [39] subjected the players to endurance tests to exhaustion on a cycle ergometer, showing that the time to exhaustion was significantly reduced at 35 °C compared to 22 °C. Additionally, all aerobic endurance indicators (oxygen uptake, ventilation, heart rate, and lactic acid) were significantly affected. Similarly, the research of Edwards et al. [55], conducted on soccer players using a field test (YoYoTEST), showed that a 2% body weight loss was harmful in endurance tests to exhaustion.

Beyond endurance, many studies have shown that the performance reduction in high-intensity efforts is even more significant. This was particularly evident in the study of Konefał et al. [56] based on the performance of players in the World Cup held in Brazil that same year. Specifically, the results showed that temperatures above 28 °C reduced performance (distance covered) in both moderate (*p* < 0.05) and high-intensity efforts (*p* < 0.01). The same study also showed that (i) the total number of sprints decreases at high temperatures and (ii) the development of maximum speed is higher in moderate temperatures than in higher temperatures. In contrast, there was no statistically significant difference in the total distance covered by the players depending on the environmental temperature, indicating that high temperatures have a greater impact on high-intensity actions than endurance.

However, these findings contrast with other studies. Özgünen et al. [57] showed that the total distance covered by soccer players during a match significantly decreases in the second halves of games played in high-temperature conditions compared to the total distance covered in the second halves of games played in moderate temperatures. Aughey et al. [58] concluded that players in high temperatures reduced low-intensity actions to maintain the ability to engage in high-intensity activities, which typically determine the outcome of matches. It seems that players employ various strategies to decrease the negative consequences of high temperatures on their physical activity [46,58].

There are also impacts on players’ cognitive functions, as high heat exposure can affect the central nervous system. Soccer is a sport where tactics play a crucial role [59]. Brain functions such as visual perception, attention, anticipation, and memory are essential for decision-making and necessary for executing correct tactical actions. Fortes et al. [43] showed that dehydration in soccer players increased the rate of incorrect choices in making the most appropriate pass, which can determine the outcome of an entire match.

Nassis et al. [15] emphasized that heat stress not only impairs physical performance but also exacerbates physiological stress, which can negatively impact decision-making and strategic execution. In hot conditions, players often experience higher heart rates, greater fluid loss, and more unpleasant feelings, all of which contribute to diminished performance and cognitive function [10]. Heat exposure has been shown to impair soccer-specific decision-making, resulting in fewer accurate decisions and increased cognitive load [10].

Therefore, the impact of high heat exposure on performance is multifaceted, affecting endurance, high-intensity efforts, and cognitive functions. As a result, effective management of hydration and acclimatization is essential to mitigate these adverse effects and maintain optimal performance levels in soccer players exposed to hot conditions.

### 4.3. Strategies to Prevent Negative Consequences

#### 4.3.1. Hydration

Preventing dehydration is a primary objective when aiming to mitigate the negative consequences of exercising in high environmental heat. Inadequate hydration strategies before, during, and after sports activities can significantly impact both athletic performance and the health of athletes [37]. Edwards et al. [55] highlighted that the negative effects on performance are not due to the sensation of dry mouth but to the actual fluid loss. Another study showed that players often begin a match with some degree of dehydration and that they may not consume adequate amounts of liquids during the match, therefore ending the match even further dehydrated [9]. This indicates that hydration advice and protocols should encompass the time before, during, and after a match or training session to effectively ensure euhydration.

Hydration guidelines indicate consuming 150–350 mL of fluids every 15 to 20 min of exercise in the heat, but individualization is crucial due to significant differences in sweating rates among individuals as well as due to the need to tailor hydration plans to the environmental conditions [33]. Clubs should tailor hydration strategies to minimize performance reduction risks. Djaoui et al. [36] noted that hydration needs vary by player position, while Francescato et al. [37] found that when athletes drank water at will, men drank less than needed, unlike women who consumed adequate amounts. Thus, athletes’ hydration status should be monitored before and after games using various methods such as plasma osmolality, urine osmolality, urine color, urine specific gravity, and body weight measurements [60]. Special attention should be given to pre-training or pre-game hydration, especially with double obligations within 24 h [9,33].

Sports drinks containing 6% glucose or a 10% glucose polymer solution significantly improve performance in repeated sprints compared to plain water [38]. Sports drinks may stimulate more intake due to their taste compared to tasteless water [9]. Furthermore, when combined with high sweat rates, excessive plain water consumption can lead to hyponatremia [37]. Care should be taken with fluid amounts and concentrations to avoid gastrointestinal discomfort, especially in high-intensity exercise where gastric emptying is limited [33].

#### 4.3.2. Heat Acclimatization

Teams acclimatized to high temperatures have an advantage over non-acclimated ones when competing in hot environments. A 5–7 day acclimatization period can improve performance significantly. Buchheit et al. [61] showed that after the first two days, the internal-to-external load ratio decreased until the fifth day and remained stable until the 8th day. Clear signs of heat acclimatization, such as improved cardiovascular capacity and neuromuscular function, were evident after 8 days. An earlier study by Buchheit et al. [62] indicated that one week of acclimatization improves exercise heart rate (i.e., decreases) and heart rate variability (i.e., increases). In 5–7 day protocols, plasma volume increases by 4–6%, while in 7–12 day protocols, the increase is about 7–13%. An increase in plasma volume enhances the body’s fluid reservoir, supporting increased sweating and facilitating cooling, thereby reducing core temperature [63].

Six-day acclimatization can achieve a 34% increase in sweating rate and an 18% reduction in [Na^+^] in sweat, preventing increased Na^+^ loss post-acclimatization despite increased sweating [64]. Significant individual differences exist in the acclimatization response. Racinais et al. [64] found that players with the most substantial hematological adaptations maintained similar activity levels in the heat compared to normal temperatures. However, there are non-responders who may not acclimatize as effectively. Pethick et al. [63] also showed large differences in plasma volume increase among individuals. Elite athletes close to maximum plasma volume limits may need fewer days for acclimatization. Thus, while a week is generally enough for acclimatization, the extent of the response varies significantly. Team medical and coaching staff should conduct pre- and post-acclimatization tests to predict players’ capacity to handle high temperatures competitively [64].

#### 4.3.3. Additional Strategies and Insights

Researchers are exploring additional methods to mitigate the effects of high heat exposure during exercise. Studies by Price et al. [65] and by Parris and Tyler [66], who used ice vests, showed beneficial effects on core temperature, perceived fatigue, and heat sensation, particularly in the second halves of soccer matches, without improving maximum running speed. Further research is needed to study the effects on other motor and cognitive abilities.

The research of Coull et al. [44] indicated that tyrosine intake is associated with increased alertness and cognitive function during exercise in high heat without improving physical performance. The pharmacokinetics of tyrosine and its long-term effects on health and performance require further study. Additionally, No and Kwak [39] found that bupropion administration improved performance in heat by enhancing norepinephrine and dopamine action, which regulates mood, motivation, and energy.

Nassis et al. [15] highlighted several strategies for managing heat stress, such as adjusting warm-up durations and structures, using cold water immersion during halftime, and applying ice towels. These methods aim to reduce internal body temperature and improve physiological function. Combining pre-cooling and mid-cooling techniques, improving hydration, and focusing on thermal sensation during cooling breaks are also suggested. The inclusion of medical personnel in decision-making processes, particularly in match scheduling, is crucial for safeguarding player health.

Benjamin et al. [14] emphasized the importance of maintaining euhydration (i.e., optimal hydration) and using ice-water dousing to cool down, enhance performance, and manage physiological strain during exercise in hot conditions. This practical, cost-effective method offers additional performance benefits, particularly in intense, intermittent exercise scenarios. A recent study showed that athletes exercising in the heat with a core body temperature of 39.5–42.8 °C should be immersed for 11–12 min when the water temperature is ≤9 °C and for 18–19 min when the water temperature is 10–26 °C to return to a safe core temperature of 38.6 °C. Shorter immersions should be used for athletes with lower core body temperatures [67].

Finally, Donnan et al. [13] and Donnan et al. [10] investigated the impact of exercise-induced fatigue and heat exposure on soccer-specific decision-making and physical performance. The findings underscore the importance of preparing athletes for high-heat conditions to maintain optimal performance and reduce the risk of decision-making errors during competition. Practical recommendations include balancing physical exertion to sustain quick and accurate decision-making throughout the match.

Therefore, preventing dehydration, effective heat acclimatization, and innovative cooling strategies are crucial for maintaining soccer players’ performance and health in high-heat conditions. These strategies, supported by an appropriate balance of physical exertion, help mitigate the adverse effects of heat on players, ensuring better performance and overall health.

## 5. Limitations

Although the documents included in the narrative review are extensive, a limitation is the lack of a systematic approach in the review process. Additionally, due to the methodology of the bibliometric analysis, it is possible that it did not include studies that are not present in Scopus but are present in other databases. Also, because the BOOLEAN expression required specific words in the title related to high heat exposure, it is possible that documents that studied environmental conditions as a whole, including heat, but without heat being their exclusive topic, were excluded. Future research could include more databases and more general BOOLEAN expressions to address these specific constraints.

## 6. Conclusions

This study aimed to contribute to the understanding of the impact of high heat exposure on the health and performance of soccer players through a narrative review and bibliometric analysis. The findings emphasize the importance of understanding how heat stress affects players and highlight the necessity of strategies to mitigate these effects. We believe this study provides useful insights for both the specialized scientific community and the broader public. It lays the groundwork for developing strategies to manage the adverse effects of high heat exposure, with the goal of ensuring the safety and performance of soccer players. For the scientific community, this study serves as a basis for further research and innovation in sports science. For coaches, trainers, and athletes, the findings offer guidance on practices that can help improve performance and protect health.

Future research exploring the interplay between tactics, biomechanics, psychology, and nutrition in the context of heat stress could provide a more comprehensive understanding of how to support athletes in high-heat environments. By addressing these areas, future studies can build on the current research, contributing to the continuous improvement of training methodologies and health protection strategies in soccer and other sports. This will ensure that athletes can perform at their best while minimizing health risks associated with high heat exposure.

## Figures and Tables

**Table 1:** Sources with at least 5 publications that are related to the impact of heat exposure on the health and performance of soccer players.

Source	Documents
Journal of Sports Sciences	8
British Journal of Sports Medicine	7
International Journal of Sport Nutrition and Exercise Metabolism	7
Journal of Strength and Conditioning Research	6
Journal of Athletic Training	6
Revista Brasileira de Medicina do Esporte	6
Scandinavian Journal of Medicine and Science in Sports	5
Journal of Science and Medicine in Sport	5
The Journal of Sports Medicine and Physical Fitness	5
Frontiers in Physiology	5

**Table 2:** Top 20 authors in citations in documents that are related to the impact of heat exposure on the health and performance of soccer players.

Author	Citations	Documents
Maughan, R	393	5
Racinais, S	379	6
Shirreffs, SM	329	5
Dvorak, J	325	4
Edwards, AM	316	2
Noakes, TD	316	2
Mohr, M	304	5
Nybo, L	263	3
Buchheit, M	252	3
Voss, SC	216	2
Binnet, M	210	3
Ersöz, G	210	3
Ozgünen, K	210	3
Aragon-vargas, LF	199	2
Mann, ME	176	1
Marfell-jones, MJ	176	1
Rankin, DM	176	1
Shillington, DP	176	1
Anderson, ML	166	2
Baker, LB	166	2

## Data Availability

The data are available at https://zenodo.org/records/13168509, accessed on 2 August 2024.
